# One-piece resection for the treatment of ventral intradural extramedullary spinal meningioma: a retrospective study

**DOI:** 10.3389/fonc.2024.1446086

**Published:** 2024-11-22

**Authors:** Guangqing Cao, Xinao Li, Dachuan Wang, Yachao Zhao

**Affiliations:** Department of Spine Surgery, The Second Hospital of Shandong University, Jinan, China

**Keywords:** ventral intradural extramedullary spinal meningioma, one-piece resection, modified McCormick functional schema, dentate ligament, spinal cord traction, dural attachment, recurrence

## Abstract

**Objective:**

This study aimed to evaluate the feasibility and efficacy of one-piece resection for the treatment of ventral intradural extramedullary spinal meningiomas (VIESMs).

**Methods:**

Between January 2017 and December 2023, all patients who underwent one-piece resection for VIESMs were retrospectively reviewed with their demographic, intraoperative and postoperative data being recorded. In addition, postoperative neurological status based on the modified McCormick functional schema (mMFS), along with radiological manifestations on the magnetic resonance imaging (MRI) were assessed and compared with that before the operation.

**Results:**

A total of 27 cases (7 men and 20 women) with an average age of 63.37 ± 10.48 years old were included in the present study with the operation time, blood loss, length of hospital stay, and follow-up periods being 292.41 ± 42.64 min, 286.85 ± 47.03 ml, 10.37 ± 1.69 days, and 16.81 ± 10.79 months, respectively. Postoperatively, one case experienced cerebrospinal fluid leakage without neurological deterioration. At the final follow-up, the mMFS scores were unchanged in seven (25.93%) cases while they improved in the remaining 20 (74.07%) cases. Finally, the MRI examinations showed that one-piece resection was successfully performed for each VIESM without a recurrence.

**Conclusion:**

One-piece resection was a feasible, safe and effective procedure for treating VIESMs. Partial removal of the ipsilateral pedicle, facet joint, and even posterior vertebral wall to establish a wide surgical corridor and vision, resection of the dentate ligaments to minimize spinal cord traction, and meticulous coagulation of the dural attachment to reduce recurrence were the key technical points.

## 1 Introduction

Spinal meningioma (SM), accounting for approximately 8% of all meningiomas but up to 45% of intradural spinal neoplasms (1, 2), represent one of the most common intradural extramedullary tumors. Typically, SMs are benign and slow-growing with the thoracic spine predominantly being the involved location (3). Generally, total excision of an SM along with its dural attachment is a long-term cure for the majority of patients complaining of local pain and/or neurological deficits (4). Currently, various surgical approaches and techniques have been proposed for treating SMs that differ dramatically depending on multiple factors such as the location and size of the lesion, anatomical level, histological subtype, spinal stability, neurological status, and complications (5, 6). Among those factors, tumor location is considered to contribute more significantly when determining the surgical approach option.

Usually, SMs are localized dorsally or dorsolaterally to the spinal cord and can be easily removed via a traditional posterior laminectomy (7). According to the pooled result of a recent systematic review, 17.06% of SMs originated from the dura ventral to the dentate ligaments (8), also called ventral intradural extramedullary SMs (VIESMs). Complete excision of a VIESM and the dural attachment (Simpson grade I) is technically challenging because of the difficulty in primary access to the lesion without spinal cord and/or nerve traction (7, 9, 10). Although efficacy in the handling of VIESMs by a variety of approaches has been reported, such as the anterior, anterolateral, posterior, posterolateral, lateral, and minimally invasive approaches, there is as yet no consensus since each has its advantages and disadvantages (11–18).

Herein, we retrospectively analyzed a consecutive surgical series of 27 patients with VIESMs to document the feasibility, efficacy, and technique aspects of one-piece resection for this disorder based on our experience.

## 2 Materials and methods

This work was approved by the ethics committee of The Second Hospital of Shandong University (approval number: KYLL2024LW025), and performed under the principles set out in the Declaration of Helsinki. Each participant signed an informed consent form to participate in this study and for the publishing of individual data and images.

### 2.1 Participants

From January 2017 to December 2023, patients who received one-piece resection for VIESMs that were diagnosed using both radiological and pathological findings were reviewed retrospectively. Exclusion criteria: 1) patients with SMs not localized ventrally to the dentate ligaments or recurrent VIESMs; 2) patients with the VIESMs not being removed via one-piece resection; 3) patients with a previous spinal surgery or follow-up time of less than 6 months. The following demographic data of the included cases were recorded: age, sex, body mass index (BMI), and follow-up durations.

### 2.2 Surgical procedures

All procedures were carried out by a senior surgeon from the same operation team. Under general anesthesia and neurophysiological monitoring, a posterior midline approach was selected to dissect the paravertebral muscles of each patient in a prone position. Then, a laminectomy was conducted to sufficiently expose the dural sac according to the cephalic, caudal, and medial margins of each VIESM. Next, the dura mater and arachnoid membrane were opened using a vertical incision. After that, one or two dentate ligaments were resected unilaterally or bilaterally from the inner surface of the dura depending on the tension of the spinal cord. By gentle rotation of the spinal cord, the exact relationship between the tumor and the spinal cord was intuitively observed. After a second assessment of spinal cord tension, the ipsilateral pedicle, facet joint, and sometimes the vertebral wall were partially removed by a high-speed drill to enlarge the operative corridor and field. Finally, the tumor with its capsule kept intact was carefully separated from the spinal cord via one-piece resection. Prior to incision closure, the dural attachment of each tumor was meticulously coagulated as much as possible to reduce recurrence. Instrumented fusion with lateral mass screws in the cervical spine and pedicle screws in the thoracic spine was performed to avoid the underlying risk of postoperative spinal instability.

### 2.3 Surgical safety

Intraoperative data including the operation time, blood loss, and length of hospital stay, along with postoperative complications such as cerebrospinal fluid (CSF) leakage, neurological deterioration, and infection were recorded.

### 2.4 Surgical efficacy

All patients were examined preoperatively and received routine follow-ups postoperatively, with their neurological functions evaluated using the modified McCormick functional schema (mMFS) before and at least 6 months after the operation (19). Meanwhile, postoperative magnetic resonance imaging (MRI) examinations were regularly done to assess the local conditions of previous lesions.

### 2.5 Statistical analysis

All data were analyzed with SPSS 20.0 (IBM Corp., Armonk, New York, USA) software and presented as means ± standard deviation for the continuous variables. The mMFS scores at the last follow-up were compared with those before the operation using the Wilcoxon signed-rank test since the deviations between them did not fit a normal distribution. It was considered statistically significant when the *P* value < 0.05.

## 3 Results

### 3.1 Patient demographics

As displayed in **Table 1**, a total of 27 patients (7 men and 20 women) who underwent one-piece resection for the management of VIESMs were included in the current study, with their ages ranging from 44 to 86 years old (mean, 70.09 ± 2.98), follow-up periods from 6 to 48 months (mean, 16.81 ± 10.79), BMI from 18.7 to 29.2 (mean, 22.73 ± 2.82), and symptom durations from 2 to 24 months (mean, 9.56 ± 5.89). Regarding the affected anatomy, cervical segments were involved in five cases (18.52%) while thoracic segments were involved in the remaining 22 cases (81.48%).

**Table 1:** Demographic data of the included cases with VIESMs.

Sex	Age (year)	Level	Location	BMI	DOS (month)	FU (month)	OT (min)	BL (ml)	HS (day)	P-mMCS	F-mMCS
Male	73	C4-5	Ventral	24.7	12	6	290	275	11	3	2
Male	58	C5	Ventral	27.4	3	12	240	400	9	2	1
Male	51	C5	Ventral	22.4	20	48	320	260	8	3	1
Female	55	C5	Ventral	21.2	6	24	240	230	10	3	2
Female	64	C5-6	Ventral	20.6	5	6	310	310	9	2	1
Female	62	T1	Ventral	23.6	5	14	315	200	9	1	1
Female	67	T1	Ventral	20.8	8	12	315	290	10	2	1
Female	60	T1-2	Ventral	22.3	7	12	250	300	14	2	2
Female	73	T2	Ventral	18.7	2	12	265	300	10	1	1
Male	58	T2	Ventral	21.6	18	36	255	300	10	2	2
Male	68	T2-3	Ventral	26.8	12	24	265	270	11	2	1
Male	64	T3	Ventral	21.3	9	36	350	250	10	1	1
Male	51	T3	Ventral	26.9	6	6	300	335	10	3	1
Female	74	T3	Ventral	20.1	4	20	300	350	13	2	2
Female	86	T4-5	Ventral	20.4	24	6	370	300	14	4	2
Female	72	T5	Ventral	22.1	10	8	350	320	12	2	1
Female	62	T5-7	Ventral	22.2	3	6	360	300	9	5	4
Female	81	T6	Ventral	19.9	12	6	230	230	13	2	1
Female	67	T6	Ventral	23.1	3	18	340	350	9	4	3
Female	67	T7	Ventral	23.1	12	12	275	260	11	3	2
Female	52	T7-8	Ventral	27.6	18	18	210	310	11	4	2
Female	58	T8	Ventral	29.2	8	12	325	250	9	3	2
Female	52	T9	Ventral	20.5	6	28	300	350	9	1	1
Female	73	T9-10	Ventral	19.6	18	12	260	200	9	2	1
Female	46	T10	Ventral	25.5	4	24	285	260	8	2	1
Female	73	T11	Ventral	21.1	11	24	320	265	12	3	1
Female	44	T11	Ventral	20.9	12	12	255	280	10	2	1

VIESMs, ventral intradural extramedullary spinal meningiomas; BMI, body mass index; DOS, duration of symptom; FU, follow-up; OT, operation time; BL, blood loss; HS, hospital stay; P- mMCS, preoperative modified McCormick Scale; F-mMCS, final modified McCormick Scale.

### 3.2 Surgical safety and efficacy

**Figure 1:**
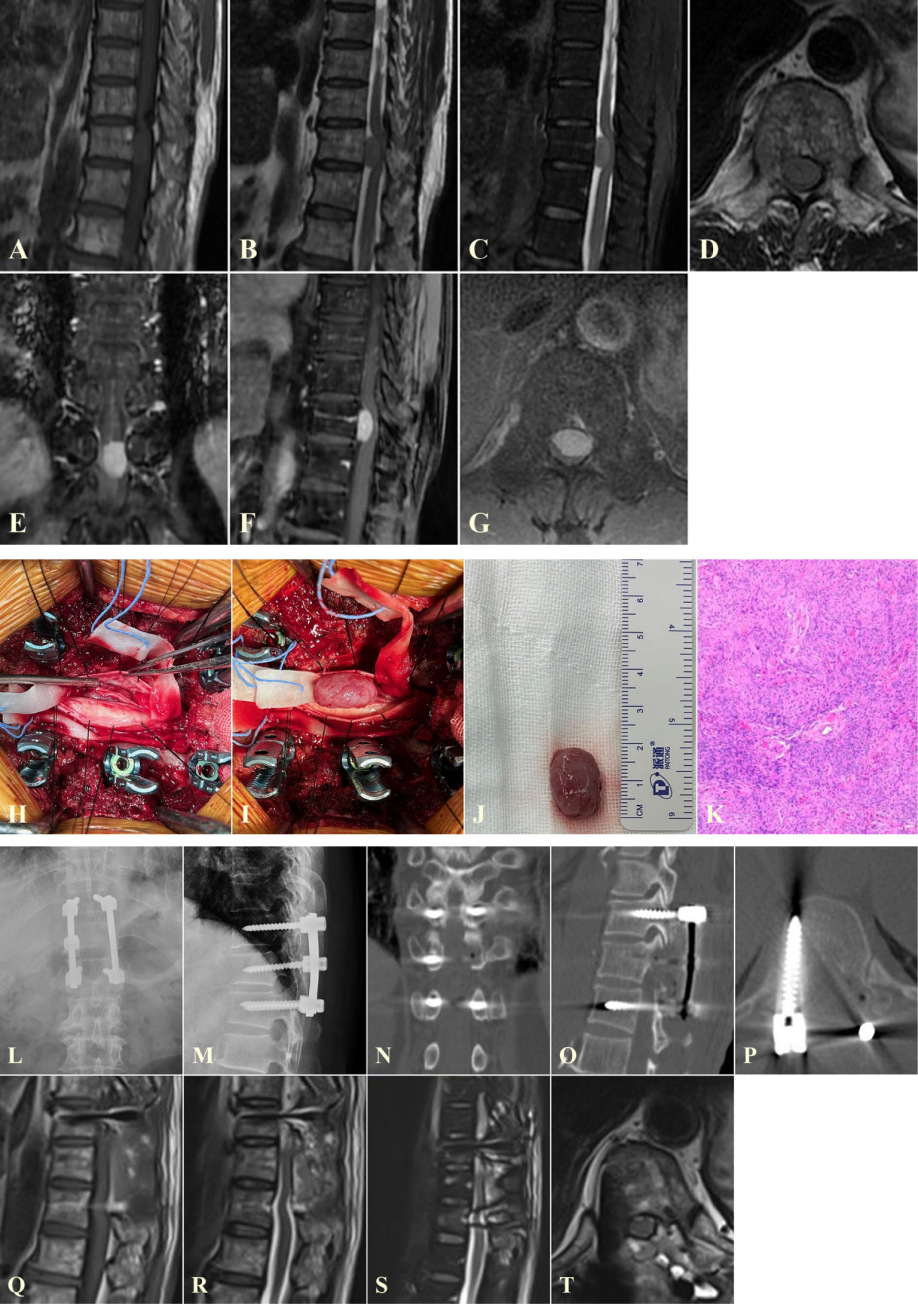
A representative case of one-piece resection for managing a VIESM at T11. **(A–G)** Preoperative MRI images showing the VIESM at T11. **(H–K)** One-piece resection of the VIESM during the operation, verified later by the pathological biopsy findings. **(L–T)** Postoperative X-ray **(L, M)**, CT **(N–P)**, and MRI **(Q–T)** images demonstrate the complete removal of the VIESM via one-piece resection and fine internal fixation. VIESM, ventral intradural extramedullary spinal meningioma; MRI, magnetic resonance imaging; CT, computed tomography.

**Figure 2:**
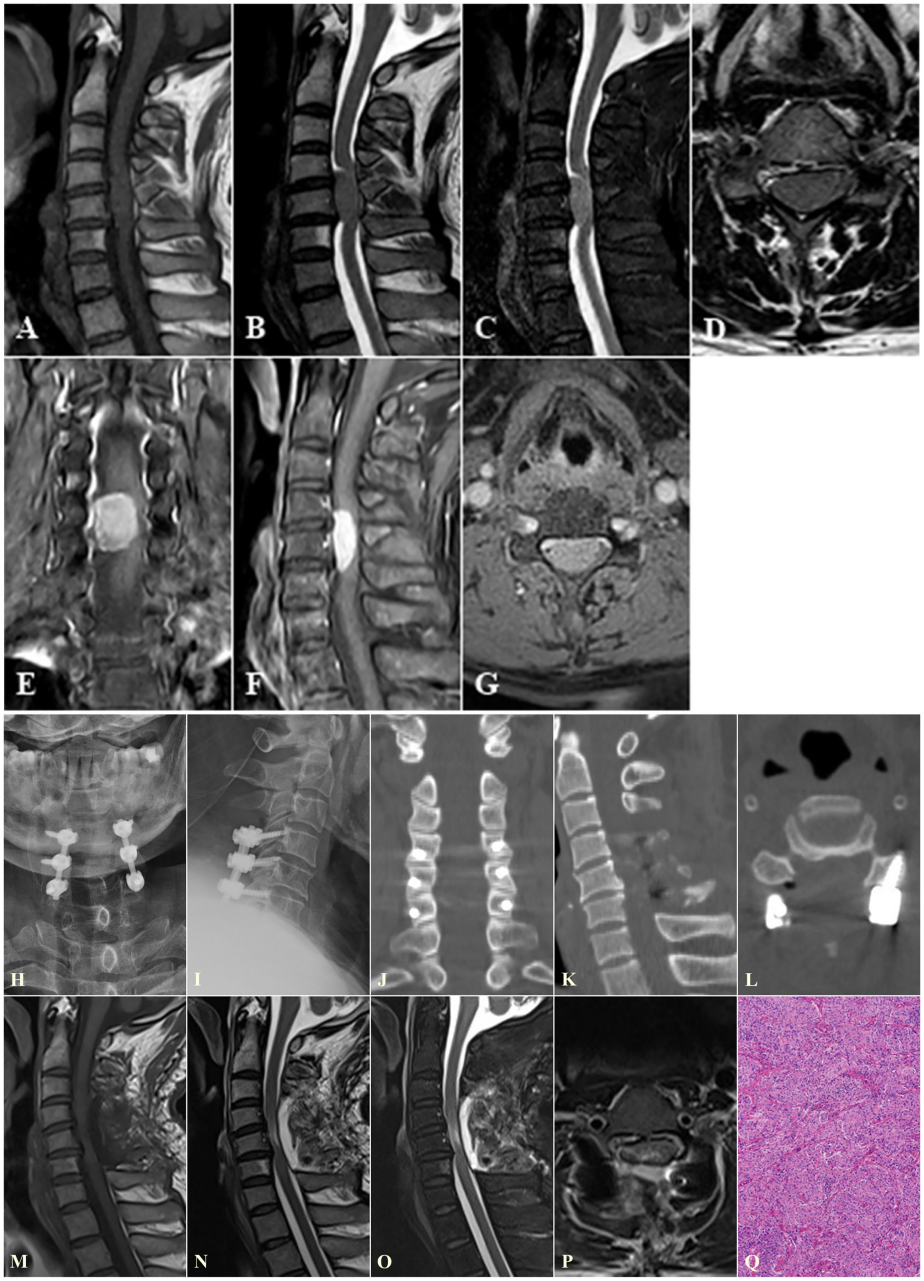
A representative case of one-piece resection for treating VIESM at C5. **(A–G)** Preoperative MRI images show the VIESM at C5. **(H–Q)** Postoperative X-ray **(H, I)**, **(J–L)**, MRI **(M–P)**, and pathological **(Q)** images demonstrate the total removal of the VIESM via one-piece resection, and fine internal fixation. VIESM, ventral intradural extramedullary spinal meningioma; MRI, magnetic resonance imaging; CT, computed tomography.

As summarized in **Table 2**, one-piece resection for VIESMs was safely carried out in all cases with their operation time ranging from 210 to 370 min (mean, 292.41 ± 42.64), blood loss from 200 to 400 ml (mean, 286.85 ± 47.03), and hospital stay length from 8 to 14 days (mean, 10.37 ± 1.69). According to the World Health Organization (WHO) classification (20), all the resected lesions were ranked as Grade I, the subtypes of which were meningothelial meningiomas in 19 cases, psammomatous meningiomas in four cases, fibroblastic meningiomas in two cases, and transitional meningiomas in two cases. Postoperatively, no neurological deterioration or tumor recurrence occurred while one case suffered CSF leakage which was then successfully treated. At the final follow-up, the functional scores based on the mMCS were improved in 20 cases (74.74%) and unchanged in the remaining seven cases (25.93%), and were significantly increased compared to those before the operation (*P*< 0.05). Representative cases of one-piece resection for VIESM are shown in **Figures 1** and **2**.

**Table 2:** Statistical analyses of the mMCS sores and intraoperative data.

mMCS		Operation time (min)	Blood loss (ml)	Hospital stay (day)
Pre-operation	Final follow-up	292.41 ± 42.64	286.85 ± 47.03	10.37 ± 1.69
2.44 ± 1.01	1.52 ± 0.75			

mMCS, modified McCormick Scale.

## 4 Discussion

As the most frequently seen intradural benign tumor, SM often needs a surgical excision to prevent its progression and further compression to the neighboring spinal cord or nerves (21). To lower the risk of SM recurrence, complete tumor removal is of great necessity during initial resection. Nevertheless, it remains a difficult surgical challenge to handle SMs located ventrally to the dentate ligaments, namely VIESMs, due to the limited operative field and high risk of spinal cord traction (22). In this study, we presented a consecutive series of patients diagnosed with lower cervical or thoracic VIESMs, and shared our experience regarding one-piece resection for those ventral lesions. Among the included cases, the majority of VIESMs were located in the thoracic spine (81.48%), which was consistent with previous reports (23, 24).

Although several studies exhibited satisfying results (6, 9, 11, 15, 16), no current consensus has been reached on which approach is better for VIESM resection when taking the lesion size, affected segment, spinal stability, and complications into considerations. As indicated, the anterior or anterolateral route facilitated direct access to the lesions, but it provided a limited surgical field and narrow working corridor albeit with increased bone removal and increased risk of CSF leakage and injuries to anterior structures (11, 12). Conversely, the posterior or posterolateral approach offers excellent operation vision and space while primary access to the ventral lesions is quite difficult without any spinal cord traction (5, 13, 14). Few reports applied a minimally invasive approach to VIESM resection and the indications were mainly restricted to small lesions with no dense adhesion (18, 25). Additionally, the lateral approach allowed adequate lesion exposure while avoiding excess spinal cord manipulation, but this unfamiliar approach has a learning curve and does not allow for the insertion of lateral mass screws (16, 17). In our series, one-piece resection was safely and effectively conducted for all the included VIESMs located in either the lower cervical or thoracic spine by a conventional posterior route. At the last follow-up, no neurological deterioration or tumor recurrence occurred, which suggests the feasibility of total VIESM removal through this approach.

Considering the issues involved in direct access to the ventral lesions and their total excisions by a posterior approach as described above, the ipsilateral pedicle, facet joint, and sometimes the vertebral body were partially removed following a laminectomy to create a wide corridor with sufficient surgical vision for the subsequent resection of VIESMs. Similar to other reports, to minimize spinal cord traction (5, 11, 14, 26), we cut off the dentate ligaments unilaterally or bilaterally depending on the tension of the spinal cord and no neurological deterioration happened postoperatively. Although Kiyoshi et al. (6) demonstrated access to ventral thoracic spinal tumors by the removal of the unilateral pedicle and facet joint without posterior fixation, long-term follow-ups were still required to assess postoperative spinal stability. To avoid the potential risk of secondary instability and support in the early mobilization period, we used posterior instrumentation in each patient following one-piece resection. Preserving tumor integrity allows for ascertaining lesion origin and its attachment, thus decreasing the recurrence rate (27). While the complete removal of lesions and the attached dura (Simpson grade I) can prevent recurrence, it is difficult and elevates the risk of CSF leakage if they are located ventrally (28). As indicated by King et al. (29), excision of the dural attachment was not necessary for lowering SM recurrence. Instead, a Simpson grade II resection of SMs was acceptable and associated with a low recurrence rate (28, 30). We also conducted Simpson grade II resections for all the VIESMs with the dural attachment treated by meticulous coagulation. As a result, no recurrence was found and most of the patients (74.07%) presented with satisfying outcomes at their last follow-up, indicating the feasibility and efficacy of this procedure. During this study period, all VIESMs were successfully removed via one-piece resection. However, we hold the opinion that if the tumor is located ventrally to the cervical spinal cord and is too large, it may not be suitable for one-piece resection as there is a risk of neurological deterioration and overstretching the nerve root when reducing the tension of spinal cord. 

This retrospective study has some limitations. First, it was a case series study with a small sample size, which is mainly attributed to the rarity of VIESMs. Second, follow-ups should be continued to observe the long-term safety and efficacy of this procedure for handling a VIESM. Third, there is no control group with VIESMs treated by other strategies, which reduces the robustness of evidence offered by the present study.

Taken together, one-piece resection was a feasible and effective procedure for managing VIESMs. Here, the partial removal of the ipsilateral pedicle, facet joint, and even posterior vertebral wall to establish a wide surgical corridor and vision, the resection of the dentate ligaments to minimize spinal cord traction, and meticulous coagulation of the dural attachment to reduce recurrence were the key technical points. High-quality studies with large samples and long-term follow-ups are needed to offer more robust evidence on this topic.

## Data Availability

The original contributions presented in the study are included in the article/supplementary material. Further inquiries can be directed to the corresponding author.
